# Law, Ethics, Religion, and Clinical Translation in the 21st Century—A Discussion with Andrew Webster

**DOI:** 10.1002/stem.519

**Published:** 2010-09-15

**Authors:** Majlinda Lako, Alan O Trounson, Susan Daher

## About Professor Andrew Webster

Professor Webster received his B.Sc. in Social Sciences in 1974 at the Polytechnic of the South Bank, then earned his Ph.D. in the Sociology of Science at the University of York in 1981. He is currently Professor in the Sociology of Science and Technology, Director of the Science and Technology Studies Unit (SATSU), and Academic Coordinator for the Social Sciences at the University of York.

From 2005 to 2009 Professor Webster was the Coordinator of the International Stem Cell Initiative (SCI), and is currently the Coordinator of the Regenerative Medicine in Europe (REMEDiE) project as well as a member of the Medical Research Council's UK Stem Cell Bank Steering Committee, the Medical Research Council's Medical Ethics Committee, the UK National Stem Cell Network Committee.

Dr. Webster sits on the editorial board of the journals *Health Informatics*, *New Genetics and Society*, and *Industry and Higher Education*. He was elected a Fellow of the Academy of Social Sciences in 2007.

## “As a Sociologist, I Have Always Been Interested In The Social, Cultural, And Economic Factors That Shape Health Innovation…”

“The development of new health technologies has long been a focus of my research since completing my doctorate at York almost 30 years ago. As a sociologist, I have always been interested in the social, cultural, and economic factors that shape health innovation, especially in respect to new developments in the biosciences (my earliest work was on the radical changes brought about by molecular biology in the mid-1980s). My work has shown how new health technologies and the techniques, models, and assumptions on which they are based are tied into other technologies, practices, and social relations and how their adoption depends on the way these processes work together. This is true whether they appear in the most mundane (such as the stethoscope) or the most advanced (say the magnetic resonance imaging scanner or human embryonic stem cells [hESCs]) of forms. This has important implications for the perceived utility and so translation of innovation into practice.”

“Over the past 10 years, I have directed two major research programs exploring these issues, the Innovative Health Technologies program and the Stem Cell Initiative, funded by the two research councils, the Economic and Social Research Council (ESRC) and the Medical Research Council (MRC). The first covered a wide range of innovation, including pharmaceuticals, new devices, and ehealth, while the more recent has been exclusively devoted to stem cells development in the UK and more globally.”

## “My Work at York Has Focused on Standardization of hESCs”

“The standardization process has been marked by a gradual stabilization of some biological markers for hESCs, but these have not proved definitive in terms of ‘stemness’ and so ‘pluripotency,’ nor predictive of patterns of differentiation into specific cell lines. We have found that the problem of determining pluripotency has led to a growing pragmatism in the hESC network to continue to produce what are seen as productive results, effectively a shift from a search for ‘essential’ to ‘functional’ attributes of hESCs. Standardization has become to be seen as meaningful and so useful through a focus on ‘how’ rather than ‘why’ lines do what they do. This suggests hESCs are less ‘pluri’ but (ironically thereby) more ‘potent’. Standardization is of course double-edged inasmuch as it can also impose a method on the community that may drive science toward early, suboptimal models of biological markers. Moreover, even if this were not an issue, the goal to create general reference standards across laboratories is difficult to achieve because it is often the case that the way cells work in laboratories differs when they go into good manufacturing practice (GMP) ‘clean rooms.’ We have also examined how standardization relates to the automation of cell cultures and scale-up, and have argued that in respect to the latter there's an important role to be played in the near future, probably by a public sector or not-for-profit body, doing some of the basic spadework, especially in regard to purification and scale-up, for the wider community.”

“These particular interests we have in the field reflects one of the core themes of the Science and Technology Studies Unit (SATSU) at York, that is, the sociology of the biosciences. Our work has examined not only developments in regenerative medicine (RM) but also the development of pharmacogenetics, bioinformatics, and cord blood banking. The unit also focuses on two other research themes: the regulation of new science and technology and the role of the digital world in shaping social life more generally.”

**Figure 1:**
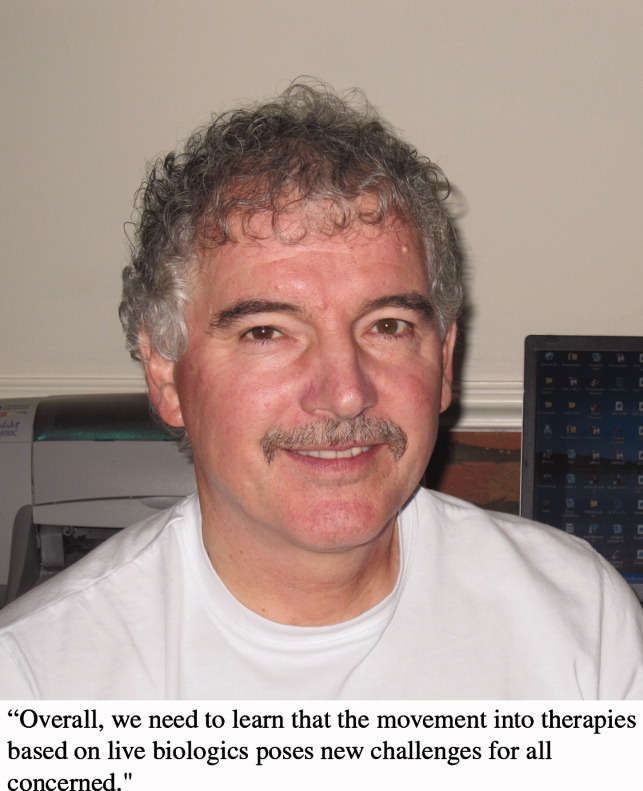
Andrew Webster, Director, Science and Technology Studies Unit, University of York.

## “Many Sociologists Work Closely, As I Have Done, With Stem Cell Scientists And Clinicians On Shared Concerns And Interests”

“For example, I have been involved in collaborative work with the Centre for Stem Cells Biology on how standards have been developed internationally across the hESC community and the technical and transorganizational issues that have had to be addressed. Another collaboration with colleagues at York has explored the innovation paths that are more, or less, successful in respect to hematopoietic cell lines. The SCI provided an excellent opportunity to explore the social, cultural, regulatory, and economic factors that shape the development of RM, enabling a strongly comparative analysis involving research in Europe, the U.S., China, Japan, and India. It is clear from the findings that the status and role of RM varies hugely across countries and this is only partly linked to political or religious beliefs. Much also depends on the more important relationship between research and clinical systems and the role and relative impact of international and local regulatory agencies. In Japan, for example, there is a very strong clinical push in tissue engineering that depends on the government promoting tight relations between hospital clinicians and researchers (with little room for corporate activity).”

## “…There Is a Need To Define What The Quality Control Requirements Of Large-Scale Cell Culture Are Likely to be Before The Move Toward Securing Clinical Grade Lines Is Made”

“My role as Coordinator in the SCI began in November 2005 and ran through to June 30, 2009. The ESRC has provided £3 million to support a range of activities that would build research capacity and raise awareness within the UK social science community in regard to the emerging field. The SCI network has now grown to some 45 UK-based researchers who have focused on a number of discrete areas, such as the development of standards in the field (crucial to its longer term success), the pattern of capital investment and return it has, the clinical and ethical questions it raises, and what new issues it poses for those charged with regulating an ever-changing and at the same time uncertain ‘promise’ of product development and clinical therapy. The SCI membership has been drawn on by a wide range of stakeholders, especially within the policy and bioscience arenas, to provide advice and input to a wide range of debates and specific policy questions, such as the development of the HFEA Bill in 2007–2008, the work of the UK Stem Cell Bank, the Stem Cell Dialogue project run by the MRC and BBSRC, close cooperation with the UK National Stem Cell Network, and wide engagement with divergent publics within the different regions across the UK, both during and beyond the annual ‘Science Weeks.’”

“The REMEDiE project, that I coordinate, examines the current European engagement with RM and how this compares with that found more globally. Its primary objective is to understand how the political, economic, and bioethical developments within the field operate at national and transnational levels and shape the future clinical and corporate activity within RM. The various Work packages focus on different aspects of these three areas and undertake comparative empirical analyses within and beyond Europe. The overall objective is to determine how the project findings can contribute toward European Community Member State and European Parliamentary policy-making in this area. In regard to the latter, the Consortium has liaised with the Scientific Technology Options Assessment group, the European Parliamentary Agency that advises on future technology options, and is providing information about the results of our work this coming November. We have also linked up with the European Patients Foundation to provide results through their Newsletter. Some of the current work we are doing is showing that in regard to the RM science base, Germany and the UK have the stronger national research capacities in the field (the latter with notably strong links across the social and biosciences) and have the potential to move more quickly in this area in terms of innovation, effective regulation, and clinical delivery. Germany is also particularly well placed in regard to the financial stability of its RM investment profile compared with other Member States.”

“On the question of clinical translation two key points are emerging: the prospective take-up of new products will depend on firms addressing more effectively matters of clinical utility and relevance against existing therapies or products, if they are to persuade end users of the relative merits of RM. Second, there is a need to define what the quality control requirements of large-scale cell culture are likely to be before the move toward securing clinical grade lines is made. This will require dialogue between regulators, stem cell banks (such as those in the UK and Spain), clinical research laboratories and companies.”

## “What Is Important Is How The Regulatory Environment Approaches The Variability of Any Lines as They Are Scaled Up…”

“The most recent developments in RM are often said to be associated with the arrival of induced pluripotent (iPS) cells. Although these short-circuit much of the ethical debate of hESC-derived lines, and have led to a huge increase in big Pharma interest, the stability of such lines in terms of therapeutic development is as tricky an issue as it is for hESCs. How much biological variability can be accommodated while GMP variability is not? Another key issue relates to the ways in which current clinical trials in the field are working to police and manage it effectively and whether any changes in trial protocols because of the problems of working with biologically live tissue are needed. Both these issues raise questions about the sort of longer term equivalent of pharmacovigilance that will be needed to optimize patient safety in the RM field.”

“There is a lack of an internationally unified regulatory framework for stem cell and tissue transplantation (which creates various problems such as the proper management of the transfer of hESC lines between different regulatory jurisdictions). Problems with inconsistent tissue banking procedures, inconsistent donor consent, uncertain efficacy and discordant regulatory standards make things especially problematic.”

## “Trust In Science Is a Recurring Theme In This Research, for Both Scientists and the Public”

“In general, public opinion has been broadly very favorably disposed to stem cell research as the Stem Cell Dialogue report found. Extensive work by one of the SCI teams (Sarah Parry and her colleagues at the University of Edinburgh) on public attitudes to stem cell science shows that most people did not object to the use of human material such as cord blood, embryos, or fetal tissue, even if they might express some discomfort about this. An important intermediary agency in helping to reassure public opinion has been the UK Stem Cell Bank which plays a pivotal role as a public-good agency in helping to ensure that the sourcing and management of cell lines is conducted according to a high ethical standard.”

“Much of the Stem Cell Dialogue was about the conventional approach that says we need a better public understanding of science. Although this is always to be fostered, it has long been demonstrated that more understanding does not thereby yield more (unquestioning) support for the field. Rather it leads to a more nuanced and perhaps critical view. Rather than seeing this as a problem, it would be much better to be more ready to acknowledge the provisionality of the science and thereby the prospects for therapy. It would be much better to engage with the public in terms of a ‘public understanding of scientific practice’ which would seek to show the challenges that are faced on the ground. This would provide for a more informed engagement with science and between science and the diverse publics ‘out there.’ It would also help to avoid the litigious nature of the US stem cell policy process which most recently has seen a judicial ruling bringing a halt to Federal funding of hESC research. Even so, government and the science community need to be clear about where they see the line being drawn with respect to actual public engagement in wider governance processes.”

## “The Eventual Business Model for the New Generation of RMs Based On Either iPS or hESC-Derived Therapies Will Crucially Depend on the Practical Context In Which The Technology Can Be Used”

“This business model is very likely to be much more limited than some of the hype one reads, and more likely to involve application in surgical settings rather than as a therapy akin to a mass drug market. On the other hand, what we are seeing in China may break the mold through structuring hospital/research laboratories relations much more tightly together, allowing for a wider adoption of RM there. In the U.S. and Europe, much will also depend on companies ensuring follow-up for patients after delivery of their products. For example, Apligraf was only successful after the specification for the product was changed in response to patient/carer feedback. This highlights the importance of clinical context. Two things need to be understood here: first, clinical relevance, what would make something worthwhile having? Second, clinical practice, what organizational and cultural factors determine this?”

“Over the next 10 years or so there will be some key issues that will need to be followed. These include the iPS/hESC relationship and how this will play out in not merely a technical sense (determining pluripotency for example) but a regulatory one too (in respect to future trials and questions of safety). In regard to trialing itself, the outcome of both the UK ReNeuron and US Geron trials will be especially important. Clearly, it is in Geron's interests that their trial is clinically successful to ensure their stock, both in its reputational and capital forms, remains high so the trial will be kept under extremely close review, by those in and outside of the company. The commercial impact of the Geron trial results is also hugely significant. If the patients begin to regain sensation but then lose it as the body's immune system kicks in, will that be regarded as a success or failure? The current volatility of the international market will in part determine how this is answered.”

“Overall, we need to learn that the movement into therapies based on live biologics poses new challenges for all concerned, and will require a quite explicit but thereby legitimate embrace of uncertainty. In a time of recession and public expenditure cuts, it is ironic that the role of public sector research will become increasingly important and play a key role in early stage clinical development. This is because translational research should be seen as a complex two-way flow of knowledge between bench and bedside, not from bench to bedside, as is the usual mantra.”

